# Telomere DNA length-dependent regulation of DNA replication timing at internal late replication origins

**DOI:** 10.1038/s41598-019-46229-1

**Published:** 2019-07-09

**Authors:** Yudai Hasegawa, Mayuko Yamamoto, Junki Miyamori, Junko Kanoh

**Affiliations:** Institute for Protein Research, Osaka University, Suita, Osaka 565-0871 Japan

**Keywords:** Chromatin structure, Origin firing

## Abstract

DNA replication is initiated at replication origins on chromosomes at their scheduled time during S phase of the cell cycle. Replication timing control is highly conserved among eukaryotes but the underlying mechanisms are not fully understood. Recent studies have revealed that some telomere-binding proteins regulate replication timing at late-replicating origins throughout the genome. To investigate the molecular basis of this process, we analyzed the effects of excessive elongation of telomere DNA on replication timing by deleting telomere-associated shelterin proteins in *Schizosaccharomyces pombe*. We found that *rap1*∆ and *poz1*∆ cells showed abnormally accelerated replication at internal late origins but not at subtelomere regions. These defects were suppressed by removal of telomere DNA and by deletion of the telomere-binding protein Taz1. Furthermore, Sds21—a counter protein phosphatase against Dbf4-dependent kinase (DDK)—accumulated at elongated telomeres in a Taz1-dependent manner but was depleted at internal late origins, indicating that highly elongated telomeres sequester Sds21 at telomeres and perturb replication timing at internal regions. These results demonstrate that telomere DNA length is an important determinant of replication timing at internal regions of chromosomes in eukaryotes.

## Introduction

DNA replication is one of the most fundamental biological processes for preserving genomic information and is initiated from multiple replication origins on chromosomes during S phase of the cell cycle. However, these events do not occur simultaneously; instead, each origin has specific replication timing that is regulated by multiple factors such as chromosomal position, location in the nucleus, and differentiation state^1–3^.

**Figure 1:**
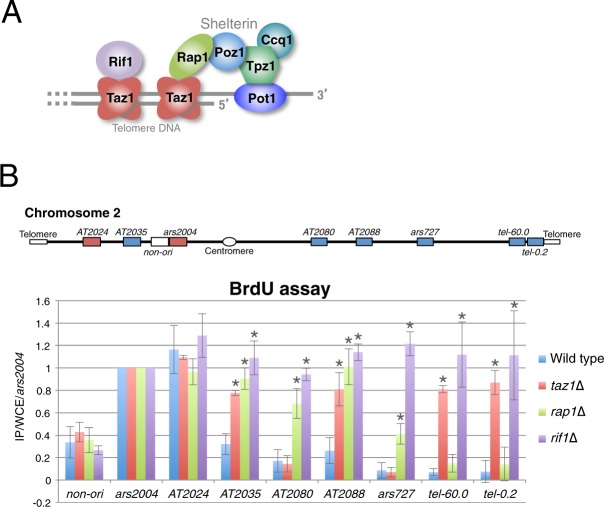
Rap1 deletion accelerates DNA replication from internal late origins. (**A**) Schematic illustration of telomere-binding proteins in *S. pombe*. (**B**) Replication timing of late origins is affected by Rap1 deletion. The locations of replication origins on chromosome 2 are schematically illustrated at the top. *AT2024* and *ars2004* are early origins (red squares), and *AT2035*, *AT2080*, *AT2088*, *ars727*, *tel-60.0*, and *tel-0.2* are late origins (blue squares). The subtelomeric late origins *tel-60.0* and *tel-0.2* are located at 60.0 and 0.2 kb, respectively, from the telomere of the right arm of chromosome 2. The non-origin region (*non-ori*) is located at 30 kb from *ars2004*. Wild type, *taz1*∆, *rap1*∆, and *rif1*∆ cells were grown in EMM medium and released from G_2_/M arrest, and their DNA was labeled with BrdU for 90 min in the presence of 10 mM HU, purified by immunoprecipitation, and analyzed by qPCR. Each value was normalized to that of *ars2004*. Error bars indicate the s.d. (*n* = 3). Asterisks indicate a significant change as compared with the wild-type strain (*p* < 0.05, Student’s t-test).

Recent studies in the fission yeast *Schizosaccharomyces pombe* have revealed the mechanisms regulating replication timing at late-replicating origins (late origins). Rif1 and Taz1, which mainly associate with telomeres at chromosome ends^4,5^ (Fig. 1A), maintain replication timing at late origins not only at telomere-adjacent subtelomeres but also at internal sites^6,7^. Rif1, the primary replication timing regulator, associates with telomeres via Taz1, which directly interacts with telomere DNA^4,5^. Interestingly, Rif1 localization near late origins is both Taz1-dependent and -independent; in the former instance, Taz1 binds to internal telomere repeat DNA and recruits Rif1 whereas in the latter, Rif1 interacts with chromatin through its affinity with G-quadruplex DNA structures^7–9^. Rif1 also directly binds protein phosphatase 1 (PP1) that counteracts the phosphorylation events by Dbf4-dependent kinase (DDK), which is essential for initiation of DNA replication^10^, to preserve the pre-replication complex (pre-RC) in an inactive state until late S phase^5,11^. Furthermore, Taz1-dependent late origins are regulated spatiotemporally during the cell cycle; they are tethered around telomeres via mutual interaction of telomere protein complexes especially during G_1_/S phase, and their replication timings are maintained at late S phase by PP1 that is recruited to telomeres by Rif1^3^.

Telomeres are specialized chromatin structures at the ends of eukaryotic linear chromosomes. Telomere protein complexes maintain genome integrity by protecting chromosome ends, regulating telomere DNA length, and tethering telomeres near the nuclear envelope^4,5,12–16^. Among *S. pombe* telomere-binding proteins, Rap1 plays a central role in the formation of telomere protein complexes such as shelterin, which bridges double- and single-stranded telomere DNAs at chromosome ends^13^ (Fig. 1A). Although recent studies have shown that Taz1 and Rif1 are important for replication timing at late origins, the precise roles of shelterin components and telomere DNA in replication timing control remain obscure.

To address this question, in this study we analyzed the effects of deleting the shelterin components Rap1 and Poz1 on replication timing. We demonstrate that excessively long telomere DNA perturbs replication timing at internal late origins but not near telomeres. Sds21, an *S. pombe* PP1, accumulated at these elongated telomeres in *rap1*∆ and *poz1*∆ cells in a Taz1-/Rif1-dependent manner, resulting in its disappearance from some of the internal late origins. Thus, telomere DNA length is an important determinant of replication timing at internal chromosomal regions.

## Results

### Rap1 deletion perturbs replication timing at internal late origins

To investigate the role of telomere-binding proteins in replication timing control, we evaluated the effect of deleting Rap1—a shelterin component (Fig. 1A) that contributes to various telomere functions in *S. pombe*^5,13,17,18^—on replication timing. We first analyzed the effect of Rap1 deletion on replication during early S phase by treating cells with hydroxyurea (HU), which depletes deoxynucleoside triphosphate pools and restricts further replication after it has been initiated at early-replicating origins (early origins) (Supplemental Fig. S1). In wild-type cells, late origins of chromosome 2 (*AT2035*, *AT2080*, *AT2088*, *ars727*, *tel-60.0*, and *tel-0.2*) did not replicate efficiently compared to early origins of the same chromosome (*ars2004* and *AT2024*) (Fig. 1B). However, internal late origins (*AT2035*, *AT2080*, *AT2088*, and *ars727*) in *rap1*∆ cells replicated with greater efficiency than those in wild-type cells, whereas replication at subtelomeric late origins (*tel-60.0* and *tel-0.2*) was largely unaffected (Fig. 1B). In contrast, deletion of Rif1—which is recruited to telomeres via association with the double-stranded telomere DNA-binding protein Taz1^4,5^—increased replication levels at all of the late origins examined while deletion of Taz1 had the similar effect at a subset of internal late origins (*AT2035* and *AT2088*) as well as at subtelomeric late origins (*tel-60.0* and *tel-0.2*) (Fig. 1B). These data demonstrate that Rap1 is required for the maintenance of replication timing at some internal late origins but not at subtelomeres, and that effects of Rap1 deletion differ from those of Taz1 or Rif1 deletion. Importantly, effects of Rap1 deletion were observed even at Taz1-independent internal late origins—i.e., *AT2080* and *ars727*, suggesting that replication timing defects at these sites in *rap1*∆ were not directly caused by disassembly of the shelterin complex.

### Excessive elongation of telomere DNA accelerates replication at internal late origins

**Figure 2:**
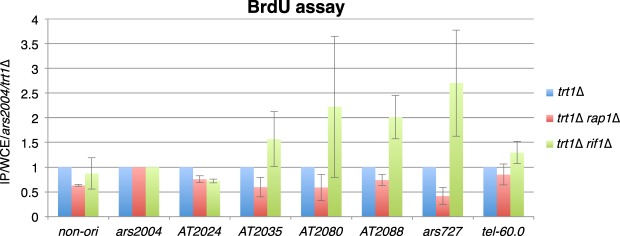
The *rap1*∆ strain shows replication timing defects in a telomere DNA-dependent manner. The *rap1*∆ *trt1*∆ strain showed replication timing similar to that observed in the *trt1*∆ strain at internal late origins in the absence of telomere DNA, whereas the *rif1*∆ *trt1*∆ strain showed accelerated replication at late origins. Cells with circular chromosomes were grown in YES medium, and the BrdU incorporation assays were performed as in Fig. 1B. Each value was normalized to that of *ars2004* and then to the *trt1*∆ value.

As described above, Rap1 is involved in the replication timing control of internal but not subtelomeric late origins; this is in stark contrast to the other telomere-binding proteins Taz1 and Rif1, the deletion of which causes defects in replication timing control at subtelomeres. Rap1 associates with Taz1 and regulates telomere length as well as various telomere functions; thus, its deletion results in abnormal elongation of telomere DNA for more than 10-fold as compared with the wild-type^5,17^ (Supplemental Fig. S2A). To explore whether absence of Rap1 *per se* or abnormally elongated telomere DNA in *rap1*∆ cells induced the observed defects in replication timing control, we analyzed the effects of Rap1 deletion on replication timing in the absence of telomere DNA. Deletion of the *trt1*^+^ gene encoding a catalytic subunit of telomerase causes gradual shortening of telomere DNA and is lethal in most cells, but some survive via self-circularization of chromosomes or recombination of chromosome end regions^19,20^. The *rap1*∆ *trt1*∆ strain with circular chromosomes and lacking telomere DNA showed no acceleration in replication timing compared to the control *trt1*∆ strain, whereas the *rif1*∆ *trt1*∆ strain showed accelerated replication at late but not early origins (Fig. 2 and Supplemental Fig. S2A,B). Thus, removal of telomere DNA from the *rap1*∆ strain suppressed the defects in replication timing. These data indicate that excessive elongation of telomere DNA and not Rap1 itself is important for replication timing control.

**Figure 3:**
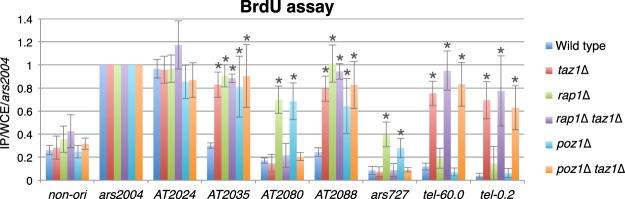
Replication timing defects in *rap1*∆ and *poz1*∆ are Taz1-dependent. The *poz1*∆ strain exhibited replication timing defects similar to those observed in the *rap1*∆ strain. These defects were suppressed by Taz1 deletion. The BrdU incorporation assays were performed as in Fig. 1B. Asterisks indicate a significant change as compared with the wild-type strain (*p* < 0.05, Student’s t-test).

We next investigated whether excessive telomere elongation caused by deletion of telomere proteins other than Rap1 also results in defects in replication timing at internal late origins. Poz1 is a subunit of the shelterin complex and as in the case of Rap1, its deletion causes abnormal telomere elongation as Rap1 deletion^13^ (Supplemental Fig. S2A). Accordingly, *poz1*∆ cells showed replication timing defects similar to those in *rap1*∆ cells—i.e., accelerated replication at the internal (*AT2035*, *AT2080*, *AT2088*, and *ars727*) but not at subtelomeric (*tel-60.0* and *tel-0.2*) late origins (Fig. 3). These data confirm that abnormally elongated telomere DNA accelerates replication specifically at internal late origins in the presence of Taz1 and Rif1.

### Replication timing defects in *rap1*∆ and *poz1*∆ are Taz1-dependent

Elongated telomere DNA has more spaces to accommodate Taz1, a protein that directly binds to telomere DNA. We therefore examined whether the replication timing defects in *rap1*∆ and *poz1*∆ cells were caused by accumulation of Taz1 at telomeres. Deletion of Taz1 in *rap1*∆ or *poz1*∆ cells suppressed the replication timing defects at Taz1-independent internal late origins (*AT2080* and *ars727*) while accelerating replication at subtelomeric late origins (*tel-60.0* and *tel-0.2*) compared to the wild type (Fig. 3). Thus, excess Taz1 bound to abnormally long telomere DNA negatively and positively regulates late replication timing at internal and subtelomeric late origins, respectively.

### Sequestering of Sds21 protein phosphatase 1 at telomeres causes accelerated replication at internal late origins

The previous data indicate that the accumulation of Taz1 at telomeres affects replication timing at internal late origins such as *AT2080* and *ars727*; a normal timing was maintained in *taz1*∆ cells (Figs 1B and 3), suggesting that Taz1 accumulation at telomeres indirectly influences replication timing at these sites. In *S. pombe*, Rif1—which binds to telomeres by interacting with Taz1 (Fig. 1A)—recruits PP1 to late origins to counteract the phosphorylation events by DDK^5,11^. Of the two *S. pombe* PP1 proteins Sds21 and Dis2, the former primarily binds to telomeres and the internal late origin *ars727* in a Rif1-dependent manner^11^.

**Figure 4:**
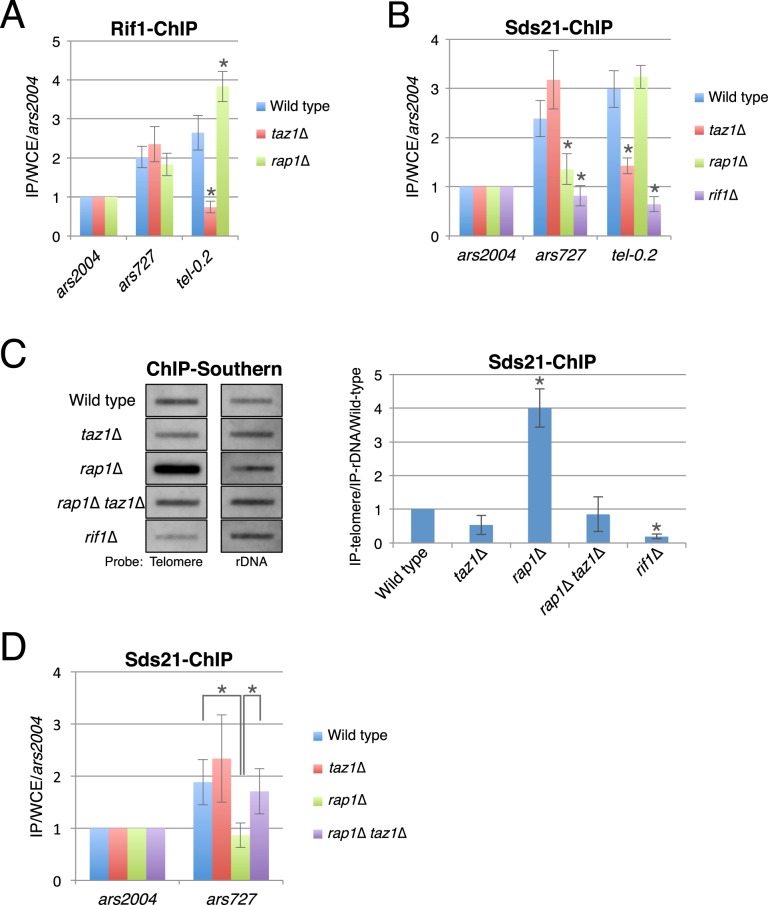
Sds21 is sequestered to excessively elongated telomeres in a Taz1-dependent manner. (**A**) Localization of Rif1 at *ars727* was unaffected by Rap1 or Poz1 deletion. ChIP analyses of Rif1-12Myc localization were performed with cells at 60 min after released from G_2_/M arrest. Co-purified genomic DNA was analyzed by qPCR. Each value was normalized to that of *ars2004*. Error bars indicate the s.d. (*n* = 3). Asterisks indicate a significant change as compared with the wild-type strain (*p* < 0.05, Student’s t-test). (**B**) Sds21 localization at *ars727* was decreased by Rap1 deletion. ChIP analyses of Sds21-3Flag were performed with cells at 60 min after released from G_2_/M arrest. Each value was normalized to that of *ars2004*. Error bars indicate the s.d. (*n* = 3). Asterisks indicate a significant change as compared with the wild-type strain (*p* < 0.05, Student’s t-test). (**C**) Sds21 accumulates at telomeres in *rap1*∆ cells. DNA co-precipitated with Sds21-3Flag was analyzed by Southern blotting using telomere and rDNA (control) probes. Left panel, representative images cropped from the same Southern blots (the original blots are displayed in Supplemental Fig. S3). Each signal compared with its background was analyzed by ImageJ software (NIH, Bethesda, MD, USA) and was normalized to that of rDNA and then to the wild-type value (right). Error bars indicate the s.d. (*n* = 3). Asterisks indicate a significant change as compared with the wild-type strain (*p* < 0.05, Student’s t-test). (**D**) Localization of Sds21 at *ars727* in *rap1*∆ cells was restored by Taz1 deletion. ChIP was performed as in (**B**). Asterisks indicate a significant change between the indicated strains (*p* < 0.05, Student’s t-test).

We examined whether the localization of Rif1 or Sds21 at late origins is altered by excessive elongation of telomere DNA caused by deletion of telomere-binding proteins (Supplemental Fig. S2A). Chromatin immunoprecipitation (ChIP) of Rif1-12myc showed that Rif1 enrichment at *ars727* was unaltered in *taz1*∆ and *rap1*∆ cells but was moderately increased and markedly reduced near telomeres (i.e., at *tel-0.2*) in the *rap1*∆ and *taz1*∆ strains, respectively (Fig. 4A). These data suggest that replication timing defects at *ars727* in the *rap1*∆ mutant is not due to reduced association of Rif1, which associates with telomeres via Taz1 as previously reported^5^.

We next examined the localization of Sds21-3Flag in the telomere-elongated mutants. In contrast to Rif1, Sds21 enrichment at *ars727* was decreased in *rap1*∆ cells as in *rif1*∆ cells, whereas no decrease was detected near telomeres (at *tel-0.2*) (Fig. 4B). Furthermore, ChIP–Southern blot analyses showed that Sds21 was enriched at telomeres in *rap1*∆ cells relative to the wild type (Fig. 4C and Supplemental Fig. S3). This accumulation was abrogated by Taz1 deletion (*rap1*∆ *taz1*∆), thereby restoring Sds21 localization at *ars727* (Fig. 4C,D).

**Figure 5:**
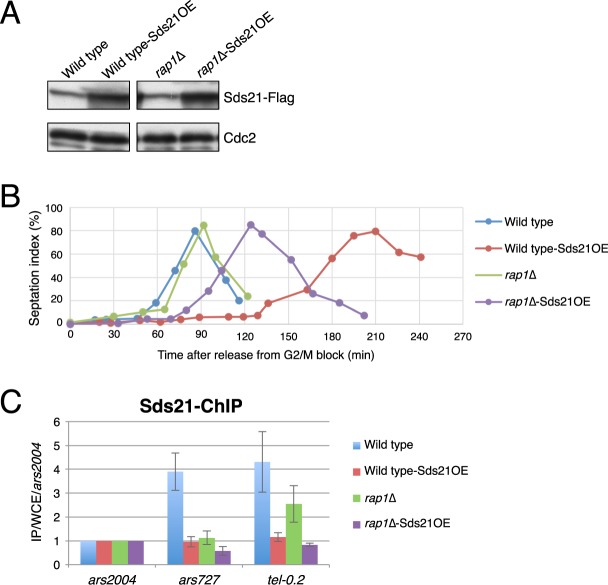
Overexpression of Sds21 causes severe defects in cell cycle progression. (**A**) Expression level of Sds21-3Flag protein in each strain. Wild type, Sds21-overexpressing wild type (wild type-Sds21OE), *rap1*∆, and Sds21-overexpressing *rap1*∆ (*rap1*∆-Sds21OE) cells were grown in EMM. The whole-cell extracts were analyzed by immunoblotting using anti-Flag (M2 F3165; Sigma-Aldrich) for Sds21-3Flag and anti-PSTAIRE (P7962; Sigma-Aldrich) for Cdc2 (loading control). (**B**) Representative septation index after release from G_2_/M block. (**C**) Wild type, wild type-Sds21OE, *rap1*∆, and *rap1*∆-Sds21OE cells were grown in EMM medium, and ChIP analyses of Sds21-3Flag localization were performed with cells in early S phase, i.e., at 60 min (for wild type and *rap1*∆), 130 min (for wild type-Sds21OE), and 80 min (for *rap1*∆-Sds21OE) after released from G_2_/M arrest. Co-purified genomic DNA was analyzed by qPCR. Each value was normalized to that of *ars2004*. Error bars indicate the s.d. (*n* = 3).

The above results suggest that the number of Sds21 molecules that can regulate replication timing is limited. To determine whether additional Sds21 proteins can rescue the enrichment of Sds21 at *ars727*, Sds21 was overexpressed (Sds21-OE) in wild-type and *rap1*∆ cells. This caused severe defects in cell cycle progression as previously reported^21^ in both wild-type and *rap1*∆ cells and decreased Sds21 localization at *ars727* (Fig. 5). One possible explanation is that excessive de-phosphorylation of target proteins by Sds21 blocks the interaction between Rif1 and Sds21. Alternatively, Sds21 may require post-translational modification for stable localization at some internal late origins, and an excess amount of unmodified Sds21 interferes with the stable localization of modified Sds21.

## Discussion

**Figure 6:**
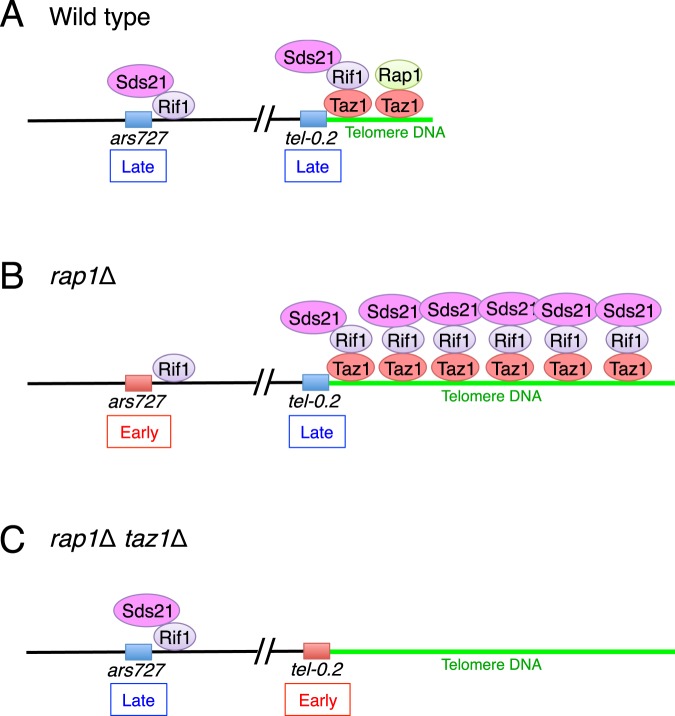
Model of replication timing control based on telomere DNA length. (**A**) In wild-type cells, the PP1 Sds21 is recruited to telomeres and late origins such as *ars727*, a Taz1-independent origin, through association with Rif1. Localization of Sds21 at *ars727* maintains replication timing at this site in late S phase. Rif1 associates with telomeres via Taz1. (**B**) In *rap1*∆ cells, telomere DNA is excessively elongated, resulting in the accumulation of Sds21 via Taz1–Rif1 complexes. Sequestration of Sds21 at telomeres reduces Sds21 localization at *ars727* and accelerates replication timing in early S phase. (**C**) In *taz1*∆ *rap1*∆ cells, Sds21 is released from elongated telomeres, which restores Sds21 localization at *ars727* and normal replication timing in late S phase. In contrast, the absence of Sds21 at telomeres accelerates replication timing at *tel-0.2*.

In *S. pombe*, Rif1 is a primary regulator of replication timing at late origins^6,7^. Rif1 recruits PP1 phosphatases (Sds21 or Dis2) to counteract the activity of DDK to maintain replication timing of late origins^11^. Rif1-dependent late origins are categorized into two groups, Taz1-dependent and Taz1-independent origins^7^. At least some of the Taz1-dependent internal late origins are tethered to telomeres by association of shelterin complexes, and their replication timings are regulated by PP1 that is recruited to telomeres by the Taz1-Rif1 complex^3^. Our results suggest that Sds21 is sequestered to elongated telomeres via the Taz1–Rif1 complex in *rap1*∆ cells, preventing its localization at some Taz1-independent internal late origins such as *ars727* and accelerating replication at these sites. The release of Sds21 from these long telomeres by Taz1 deletion restores Sds21 localization and normal replication timing at the Taz1-independent late origins (Fig. 6). Thus, we propose a new regulatory mechanism of replication timing at late origins; i.e., telomere DNA length is an important determinant of replication timing especially at Taz1-independent internal late origins.

Rif1 and Taz1 are localized at proximal regions but not at exact sites of late origins^6,7^, suggesting that PP1 that is recruited by Rif1 or Taz1-Rif1 influences a relatively broad range of late origins on chromosomes. Taz1-dependent internal late origins are clustered to telomeres especially at G_1_/S phase^3^. Our data that replication at Taz1-independent late origins (*ars727* and *AT2035*), but not at *tel-0.2*, was accelerated in *rap1*∆ cells suggest that *ars727* and *AT2035* are located far from telomeres in the nucleus. Hi-C or 3C analyses of interacting sites of chromosomes especially during G_1_/S phase will clarify spacio-temporal regulations of replication origins.

The results of this study demonstrate that telomere DNA length is a determinant of replication timing at internal late origins. In *S. pombe*, telomere DNA length is maintained by an active telomerase and the shelterin complex so the replication timing is basically unchanged over the lifespan of the organism. However, in higher eukaryotes telomere DNA becomes progressively shorter due to the decrease in telomerase activity after fertilization. Interestingly, replication timing at some chromosomal regions in mammals changes during differentiation in association with alterations in transcription levels and nuclear localization^22^. Thus, it is possible that telomere DNA-dependent replication timing control is dynamic over the course of differentiation, which is a possibility that merits further investigation.

## Methods

### Strains, media, and general techniques for *S. pombe*

*S. pombe* strains used in this study are listed in Supplemental Table S1. Experiments with *S. pombe* were performed as previously described^23–25^.

### Replication timing assay

Chromosomal DNA replication was analyzed based on bromodeoxyuridine (BrdU) incorporation as previously described^26,27^, with some modifications. Derivatives of the *cdc25-22 Pnmt1-TK Padh1-hENT* strain expressing the herpes simplex virus thymidine kinase (TK) gene and the human equilibrative nucleoside transporter (ENT) gene were grown in Edinburgh minimal medium (EMM) at 25 °C to induce TK expression and incubated at 35.5 °C for 4 h for G_2_/M arrest. (Note that the *trt1*∆ strains were grown in YES medium to maintain their cell growth.) Cells were released at 25 °C to resume cell cycle progression in the presence of 200 µM BrdU (Sigma-Aldrich) with or without 10 mM HU (Sigma-Aldrich) and were fixed with 0.1% sodium azide at pre-determined times. Genomic DNA was prepared by vigorously vortexing of the cells in S&G buffer (10 mM Tris-HCl [pH 8.0], 1 mM EDTA, 100 mM NaCl, 1% SDS, 2% Triton X-100) in the presence of phenol/chloroform, followed by ethanol precipitation. The DNA was fragmented by sonication and heat-denatured, and then used for immunoprecipitation with anti-BrdU antibody (3D4; Becton Dickinson/Pharmingen) bound to Dynabeads M-280 anti-mouse IgG (Thermo Fisher). The DNA was recovered by treatment with 1% SDS and 250 µg/ml proteinase K, and was evaluated by quantitative PCR (qPCR) with the primers listed in Supplemental Table S2.

### Flow cytometry

Cells were subjected to 70% ethanol fixation, followed by 200 µg/ml RNaseA treatment (37 °C for 4 h) and 5 µg/ml propidium iodide staining. DNA content was measured by flow cytometry of the propidium iodide-stained cells using FACSCalibur^TM^ (BD), and the data were analyzed by the CellQuest^TM^ (BD) software.

### Pulsed-field gel electrophoresis (PFGE)

PFGE of NotI-digested chromosomal DNA was performed using a CHEF-DR III Pulsed Field Electrophoresis Systems (Bio-Rad) under the following conditions: 1% SeaKem® Gold Agarose (Lonza) or Certified™ Megabase Agarose (Bio-Rad) in 0.5 × TBE; temperature, 10 °C; initial switch time, 40 s; final switch time, 80 s; run time, 18 h; voltage gradient, 6.8 V/cm; and angle, 120°.

### Southern blotting

Restriction-digested genomic DNA was separated by conventional agarose gel electrophoresis or PFGE and subjected to Southern blotting. For the telomere probe, telomeric DNA was excised from pNSU70^28^. To detect telomere-containing NotI restriction fragments of the *S. pombe* genome (designated L, I, M, and C), the *gti1*^+^, *mcp3*^+^, *fbp1*^+^, and *amo1*^+^ gene loci, respectively, were amplified by PCR as previously described^29^. To generate the rDNA probe for slot blot analyses, an rDNA fragment was amplified by PCR from *S. pombe* genomic DNA using the following primer set:

st144: 5′-CGCTAACCATTATTTACTGAGGAGAAC-3′

st150: 5′-ATCACCATATCCATATCCAATG-3′

The fragments were labeled with digoxigenin (DIG) using DIG High Prime DNA Labeling and Detection Starter Kit II (Roche), and signals were detected according to the manufacturer’s protocol.

### ChIP

Cells harboring a *cdc25-22* mutation were grown in YES medium at 25 °C to early-log phase and incubated at 35.5 °C for 4 h. They were then released from G_2_/M arrest at 25 °C to resume cell cycle progression for 60 min, fixed for 10 min in 1% formaldehyde at 25 °C and further fixed on ice for 50 min (for Sds21-Flag) or for 30 min in 3% paraformaldehyde at 25 °C (for Rif1-12myc). Crude extracts were prepared by breaking the fixed cells in lysis buffer (50 mM HEPES-KOH [pH 7.5], 140 mM NaCl, 1 mM EDTA, 1% Triton X-100, 0.1% sodium deoxycholate, 1xcOmplete [Roche]) using a Multi-beads Shocker (Yasui Kikai), after which the chromatin was sheared by sonication using Bioruptor UCD-250 (BM Equipment) to an average genomic DNA length of <0.5 kb. The insoluble fraction of the crude extracts was removed by centrifugation, and the supernatant was immunoprecipitated with anti-Flag (M2 F3165; Sigma-Aldrich) or anti-Myc (9E10; Santa Cruz Biotechnology) antibodies using Dynabeads® M-280 anti-Mouse IgG (Thermo Fisher) as a carrier. The beads were washed twice with Buffer 1 (50 mM HEPES-KOH [pH 7.5], 140 mM NaCl, 1 mM EDTA, 1% Triton X-100, 0.1% sodium deoxycholate), twice with Buffer 1′ (50 mM HEPES-KOH [pH 7.5], 500 mM NaCl, 1 mM EDTA, 1% Triton X-100, 0.1% sodium deoxycholate), twice with Buffer 2 (10 mM Tris-HCl [pH 8.0], 250 mM LiCl, 1 mM EDTA, 0.5% NP-40, 0.5% sodium deoxycholate), and twice with TE buffer (10 mM Tris-HCl [pH 8.0] and 1 mM EDTA). Immunoprecipitants and input extracts were treated with 10 µg/ml RNase A in TE buffer for 30 min at 37 °C, then with 250 µg/ml proteinase K in 0.25% sodium lauryl sulfate (SDS) at 37 °C overnight, followed by the reverse cross-linking reaction at 65 °C for 6 h. Associated DNA fragments were purified by phenol chloroform extraction and ethanol precipitation, and then analyzed by quantitative PCR (q-PCR) using StepOne^TM^ real-time PCR system (Thermo Fisher). Sequences of the primer sets for subsequent q-PCR are listed in Supplemental Table S2.

## Supplementary information

Supplemental information
