# Supplementary material for: Telomere DNA length-dependent regulation of DNA replication timing at internal late replication origins
Source: Sci Rep. 2019 Jul 9;9:9946. doi: 10.1038/s41598-019-46229-1 (PMC6617677; doi:10.1038/s41598-019-46229-1)
Supplement: Supplementary file 1 — Supplemental information [file 41598_2019_46229_MOESM1_ESM.pdf]

# Supplemental information

**for**

# Telomere DNA length-dependent regulation of DNA replication timing at internal late replication origins

Yudai Hasegawa, Mayuko Yamamoto, Junki Miyamori, and Junko Kanoh

Institute for Protein Research, Osaka University, Suita, Osaka 565-0871, Japan

Corresponding author: jkanoh@protein.osaka-u.ac.jp

Contents: Figures S1, S2 and S3

Tables S1 and S2

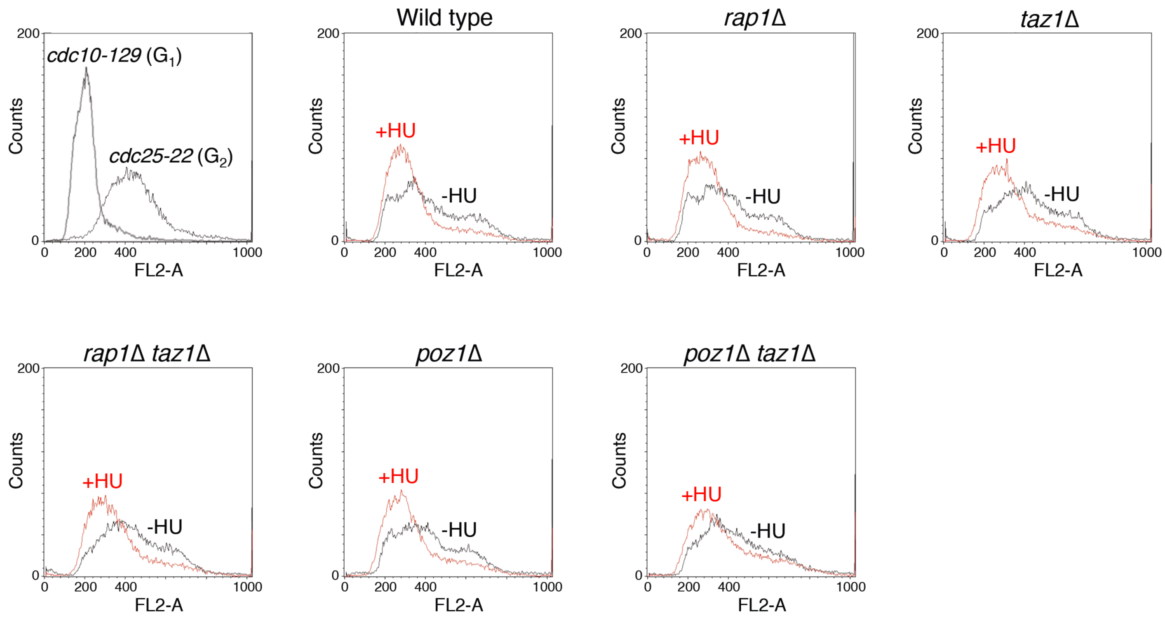


**Figure S1. Telomere mutants are arrested in early S phase by HU treatment.**

FACS (fluorescence activated cell sorting) analyses of each strain 4 h after release from the *cdc25-22* block in the presence (red lines) or absence (black lines) of 10 mM HU in EMM. X-axes indicate DNA contents per cell (FL2-A), whereas Y-axes indicate cell counts. The *cdc10-129* and *cdc25-22* strains were used as controls for 1C and 2C DNA contents, respectively.

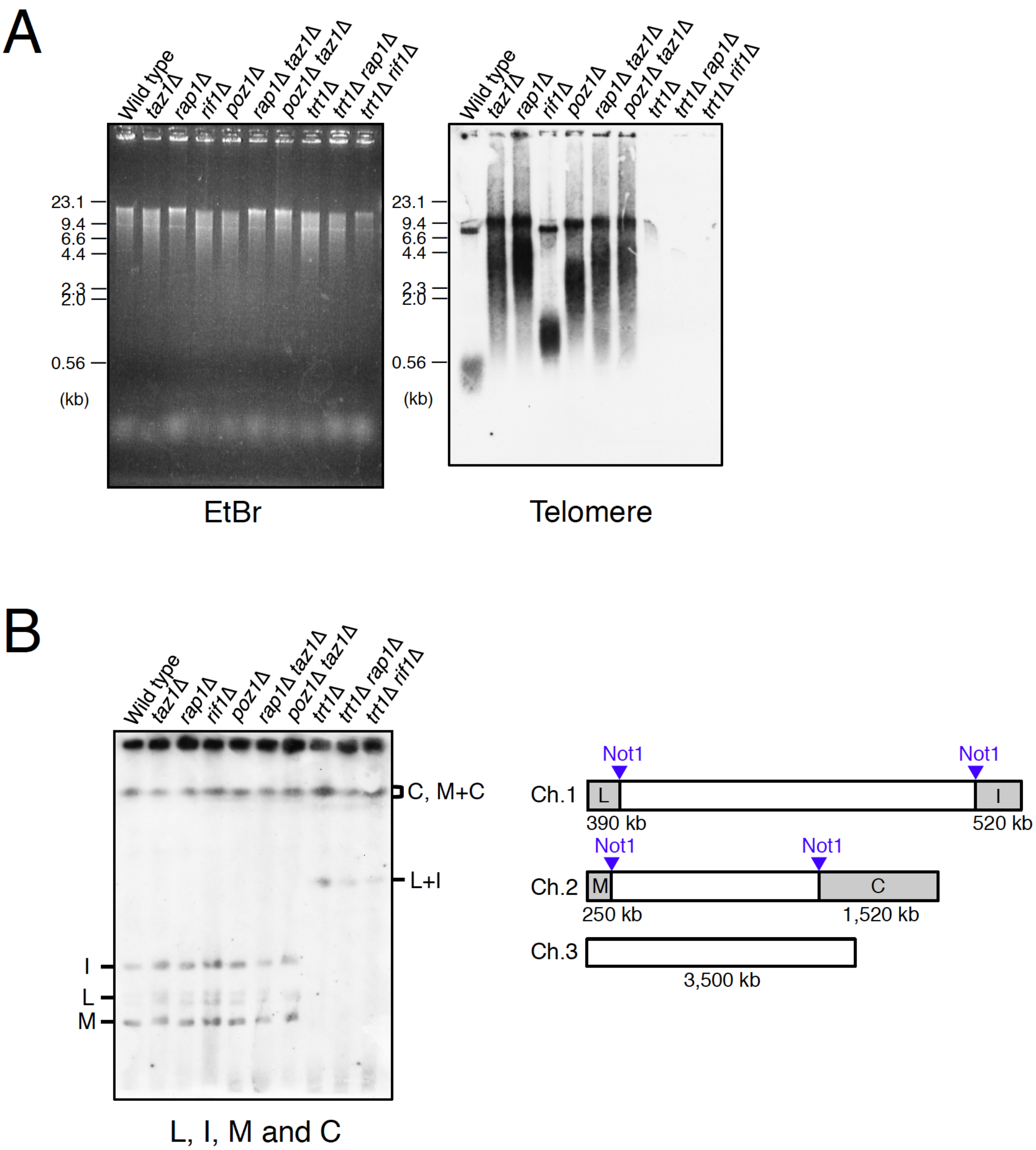


**Figure S2. Telomere DNA structures in various mutants.**

(A) Analyses of telomere DNA length. Genomic DNA was digested with ApaI and subjected to Southern blot using telomere DNA as a probe (right panel). Left panel, ethidium bromide staining of the gel after electrophoresis.

(B) Analyses of chromosome end structures. PFGE of NotI restriction fragments of the *S. pombe* genome followed by Southern blotting using the mixture of probes for L, I, M, and C fragments was performed. The right panel illustrates the telomere-containing Not1 restriction fragments in the wild-type strain.

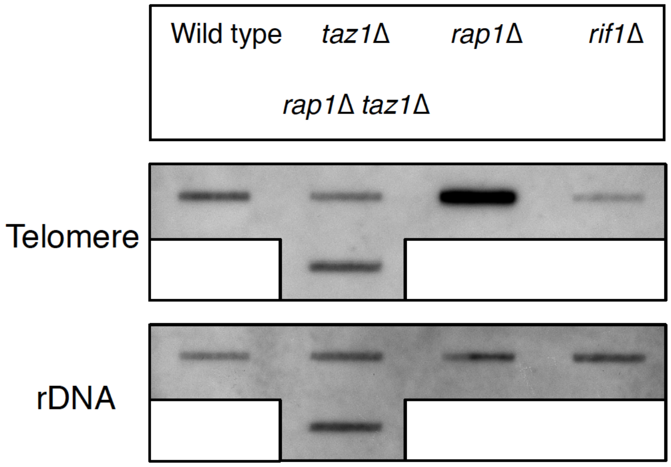


**Figure S3. ChIP-Southern analysis of Sds21.**

The original Southern blots for Fig. 4C are shown. Top, strains used in this assay; middle, Southern blot probed by telomeres; bottom, Southern blot probed by rDNA.

**Supplemental Table S1.** *S. pombe* strains used in this study

**Fig. 1C**

| | |
|---|---|
| HM1864 | *h*$^{-}$ *cdc25-22 ura4::ura4*$^{+}$*-Pnmt1-TK ade6::ade6*$^{+}$*-Padh1-hENT* (Gift from H. Masukata) |
| JP2862 | *h*$^{-}$ *cdc25-22 ura4::ura4*$^{+}$*-Pnmt1-TK ade6::ade6*$^{+}$*-Padh1-hENT taz1::hphMX6* |
| ST3167 | *h*$^{-}$ *cdc25-22 ura4::ura4*$^{+}$*-Pnmt1-TK ade6::ade6*$^{+}$*-Padh1-hENT rap1::kanMX6* |
| JP2886 | *h*$^{-}$ *cdc25-22 ura4::ura4*$^{+}$*-Pnmt1-TK ade6::ade6*$^{+}$*-Padh1-hENT rif1::natMX6* |

**Fig. 2**

| | |
|---|---|
| YH4983 | *h*$^{-}$ *cdc25-22 ura4::ura4*$^{+}$*-Pnmt1-TK ade6::ade6*$^{+}$*-Padh1-hENT trt1::kanMX6* |
| YH4985 | *h*$^{-}$ *cdc25-22 ura4::ura4*$^{+}$*-Pnmt1-TK ade6::ade6*$^{+}$*-Padh1-hENT trt1::kanMX6 rap1::hphMX6* |
| YH4987 | *h*$^{-}$ *cdc25-22 ura4::ura4*$^{+}$*-Pnmt1-TK ade6::ade6*$^{+}$*-Padh1-hENT trt1::kanMX6 rif1::natMX6* |

**Fig. 3**

| | |
|---|---|
| HM1864 | *h*$^{-}$ *cdc25-22 ura4::ura4*$^{+}$*-Pnmt1-TK ade6::ade6*$^{+}$*-Padh1-hENT* |
| JP2862 | *h*$^{-}$ *cdc25-22 ura4::ura4*$^{+}$*-Pnmt1-TK ade6::ade6*$^{+}$*-Padh1-hENT taz1::hphMX6* |
| ST3167 | *h*$^{-}$ *cdc25-22 ura4::ura4*$^{+}$*-Pnmt1-TK ade6::ade6*$^{+}$*-Padh1-hENT rap1::kanMX6* |
| JM3424 | *h*$^{-}$ *cdc25-22 ura4::ura4*$^{+}$*-Pnmt1-TK ade6::ade6*$^{+}$*-Padh1-hENT rap1::kanMX6 taz1::hphMX6* |
| YH4079 | *h*$^{-}$ *cdc25-22 ura4::ura4*$^{+}$*-Pnmt1-TK ade6::ade6*$^{+}$*-Padh1-hENT poz1::kanMX6* |
| YH4253 | *h*$^{-}$ *cdc25-22 ura4::ura4*$^{+}$*-Pnmt1-TK ade6::ade6*$^{+}$*-Padh1-hENT poz1::kanMX6 taz1::hphMX6* |

**Fig. 4A**

| | |
|---|---|
| YH4081 | *h*$^{-}$ *leu1-32 ura4-D18 cdc25-22 rif1-12myc-kanMX6* |
| YH4261 | *h*$^{-}$ *leu1-32 ura4-D18 cdc25-22 rif1-12myc-kanMX6 taz1::ura4*$^{+}$ |
| YH4257 | *h*$^{-}$ *leu1-32 ura4-D18 cdc25-22 rif1-12myc-kanMX6 rap1::hphMX6* |

**Fig. 4B**

| | |
|---|---|
| YH4491 | *h*$^-$ *cdc25-22 ura4*::*ura4*$^+$*-Pnmt1-TK ade6*::*ade6*$^+$*-Padh1-hENT sds21-3flag-kanMX6* |
| YH4493 | *h*$^-$ *cdc25-22 ura4*::*ura4*$^+$*-Pnmt1-TK ade6*::*ade6*$^+$*-Padh1-hENT sds21-3flag-kanMX6 taz1*::*hphMX6* |
| YH4495 | *h*$^-$ *cdc25-22 ura4*::*ura4*$^+$*-Pnmt1-TK ade6*::*ade6*$^+$*-Padh1-hENT sds21-3flag-kanMX6 rap1*::*kanMX6* |
| YH4497 | *h*$^-$ *cdc25-22 ura4*::*ura4*$^+$*-Pnmt1-TK ade6*::*ade6*$^+$*-Padh1-hENT sds21-3flag-kanMX6 rif1*::*natMX6* |

**Fig. 4C**

| | |
|---|---|
| YH4491 | *h*$^-$ *cdc25-22 ura4*::*ura4*$^+$*-Pnmt1-TK ade6*::*ade6*$^+$*-Padh1-hENT sds21-3flag-kanMX6* |
| YH4493 | *h*$^-$ *cdc25-22 ura4*::*ura4*$^+$*-Pnmt1-TK ade6*::*ade6*$^+$*-Padh1-hENT sds21-3flag-kanMX6 taz1*::*hphMX6* |
| YH4495 | *h*$^-$ *cdc25-22 ura4*::*ura4*$^+$*-Pnmt1-TK ade6*::*ade6*$^+$*-Padh1-hENT sds21-3flag-kanMX6 rap1*::*kanMX6* |
| YH4801 | *h*$^-$ *cdc25-22 ura4*::*ura4*$^+$*-Pnmt1-TK ade6*::*ade6*$^+$*-Padh1-hENT sds21-3flag-kanMX6 rap1*::*kanMX6 taz1*::*natMX6* |
| YH4497 | *h*$^-$ *cdc25-22 ura4*::*ura4*$^+$*-Pnmt1-TK ade6*::*ade6*$^+$*-Padh1-hENT sds21-3flag-kanMX6 rif1*::*natMX6* |

**Fig. 4D**

| | |
|---|---|
| YH4491 | *h*$^-$ *cdc25-22 ura4*::*ura4*$^+$*-Pnmt1-TK ade6*::*ade6*$^+$*-Padh1-hENT sds21-3flag-kanMX6* |
| YH4493 | *h*$^-$ *cdc25-22 ura4*::*ura4*$^+$*-Pnmt1-TK ade6*::*ade6*$^+$*-Padh1-hENT sds21-3flag-kanMX6 taz1*::*hphMX6* |
| YH4495 | *h*$^-$ *cdc25-22 ura4*::*ura4*$^+$*-Pnmt1-TK ade6*::*ade6*$^+$*-Padh1-hENT sds21-3flag-kanMX6 rap1*::*kanMX6* |
| YH4801 | *h*$^-$ *cdc25-22 ura4*::*ura4*$^+$*-Pnmt1-TK ade6*::*ade6*$^+$*-Padh1-hENT sds21-3flag-kanMX6 rap1*::*kanMX6 taz1*::*natMX6* |

**Fig. 5**

| | |
|---|---|
| YH4491 | $h^-$ cdc25-22 ura4::$ura4^+$-Pnmt1-TK ade6::$ade6^+$-Padh1-hENT sds21-3flag-kanMX6 |
| MY5543 | $h^-$ cdc25-22 ura4::$ura4^+$-Pnmt1-TK ade6::$ade6^+$-Padh1-hENT sds21::natMX6-Pnmt1-sds21-3flag-kanMX6 |
| YH4495 | $h^-$ cdc25-22 ura4::$ura4^+$-Pnmt1-TK ade6::$ade6^+$-Padh1-hENT sds21-3flag-kanMX6 rap1::kanMX6 |
| MY5545 | $h^-$ cdc25-22 ura4::$ura4^+$-Pnmt1-TK ade6::$ade6^+$-Padh1-hENT sds21::natMX6-Pnmt1-sds21-3flag-kanMX6 |

**Supplemental Fig. S1**

| | |
|---|---|
| JK2325 | $h^-$ leu1-32 ura4-D18 cdc10-129 |
| JK905 | $h^-$ leu1-32 ura4-D18 cdc25-22 |
| HM1864 | $h^-$ cdc25-22 ura4::$ura4^+$-Pnmt1-TK ade6::$ade6^+$-Padh1-hENT |
| JP2862 | $h^-$ cdc25-22 ura4::$ura4^+$-Pnmt1-TK ade6::$ade6^+$-Padh1-hENT taz1::hphMX6 |
| ST3167 | $h^-$ cdc25-22 ura4::$ura4^+$-Pnmt1-TK ade6::$ade6^+$-Padh1-hENT rap1::kanMX6 |
| JM3424 | $h^-$ cdc25-22 ura4::$ura4^+$-Pnmt1-TK ade6::$ade6^+$-Padh1-hENT rap1::kanMX6 taz1::hphMX6 |
| YH4079 | $h^-$ cdc25-22 ura4::$ura4^+$-Pnmt1-TK ade6::$ade6^+$-Padh1-hENT poz1::kanMX6 |
| YH4253 | $h^-$ cdc25-22 ura4::$ura4^+$-Pnmt1-TK ade6::$ade6^+$-Padh1-hENT poz1::kanMX6 taz1::hphMX6 |

**Supplemental Fig. S2**

| | |
|---|---|
| HM1864 | $h^-$ cdc25-22 ura4::$ura4^+$-Pnmt1-TK ade6::$ade6^+$-Padh1-hENT |
| JP2862 | $h^-$ cdc25-22 ura4::$ura4^+$-Pnmt1-TK ade6::$ade6^+$-Padh1-hENT taz1::hphMX6 |
| ST3167 | $h^-$ cdc25-22 ura4::$ura4^+$-Pnmt1-TK ade6::$ade6^+$-Padh1-hENT rap1::kanMX6 |
| JM3424 | $h^-$ cdc25-22 ura4::$ura4^+$-Pnmt1-TK ade6::$ade6^+$-Padh1-hENT rap1::kanMX6 taz1::hphMX6 |
| YH4079 | $h^-$ cdc25-22 ura4::$ura4^+$-Pnmt1-TK ade6::$ade6^+$-Padh1-hENT poz1::kanMX6 |
| YH4253 | $h^-$ cdc25-22 ura4::$ura4^+$-Pnmt1-TK ade6::$ade6^+$-Padh1-hENT poz1::kanMX6 |

*taz1*::*hphMX6*

**Supplemental Table S2. Primer sets used for quantitative PCR**

| | |
|---|---|
| ***ars2004*** | |
| yh36 | 5′-CGGATCCGTAATCCCAACAA-3′ |
| yh37 | 5′-TTTGCTTACATTTTCGGGAACTTA-3′ |
| ***non-ori*** | |
| st43 | 5′-TACGCGACGAACCTTGCATAT-3′ |
| st44 | 5′-TTATCAGACCATGGAGCCCATT-3′ |
| ***AT2024*** | |
| yh11 | 5′-GCGACGGAGATGTCCAGAAA-3′ |
| yh12 | 5′-TGCACATGCACAATACACACCTT-3′ |
| ***AT2035*** | |
| AT2035-F | 5′-TGGTACGTCGAGTGAGACACAAT-3′ |
| AT2035-R | 5′-AAAGACTCATGTGCTGTGGGAAT-3′ |
| ***AT2080*** | |
| yh15 | 5′-CGAACAACAGGCTTGGTTAGAA-3′ |
| yh16 | 5′-GAAGTACGGACTTGTTTCGATTCC-3′ |
| ***AT2088*** | |
| AT2088-F | 5′-TCAATTCTGTACGTTTCAAGCAAAT-3′ |
| AT2088-R | 5′-CCCAAATTCCACGCTGTGTA-3′ |
| ***ars727*** | |
| yh17 | 5′-TTTGCTTTACCCATGATACCCTTT-3′ |
| yh18 | 5′-CCGCGCATACCGAATGTAA-3′ |

***tel-60.0***

| | |
|---|---|
| yh13 | 5′-CAGAAGAGACTACAGAGGCGGTTT-3′ |
| yh14 | 5′-GGATGCCTTATCTGCGACCA-3′ |

***tel-0.2***

| | |
|---|---|
| jk1333 | 5′-ATTAATTGGGTAACGGAGTAACAATATAGA-3′ |
| jk1334 | 5′-CTATTTCTTTATTCAACTTACCGCACTTC-3′ |

***subtel-36 kb (from telomeres)***

| | |
|---|---|
| jk688 | 5′-CAGCCCTGGGTGCAACTATAA-3′ |
| jk689 | 5′-GAGCCCAATGACCAAAATACAAA-3′ |

***subtel-40 kb (from telomeres)***

| | |
|---|---|
| jk602 | 5′-ACGCCTTTGTTCAATCGAGTAAA-3′ |
| jk603 | 5′-CAGTGTGACTGGCACGAACC-3′ |

***subtel-46 kb (from telomeres)***

| | |
|---|---|
| jk696 | 5′-TTGAATCCCTCATCCAAAGGA-3′ |
| jk697 | 5′-TTGGTGTCAGCCCATTGAACT-3′ |

***subtel-50 kb (from telomeres)***

| | |
|---|---|
| jk606 | 5′-TTATCGCGGTGGCTATGGTT-3′ |
| jk607 | 5′-AGATCAGCCAATGGCAGATGTA-3′ |

***subtel-56 kb (from telomeres)***

| | |
|---|---|
| jk704 | 5′-TTCCAATCCCAGAGTCGTGTC-3′ |

| | |
|---|---|
| jk705 | 5′-TTTTCCCGTATGGATTGGTTG-3′ |

***subtel-60 kb (from telomeres)***

| | |
|---|---|
| jk610 | 5′-TGAGAATAAAGGGTCAGCAATGG-3′ |
| jk611 | 5′-TGTCCGTGTGGGTGAGGATT-3′ |
